# Home-based immersive virtual reality physical rehabilitation in paediatric patients for upper limb motor impairment: a feasibility study

**DOI:** 10.1007/s10055-023-00747-6

**Published:** 2023-01-14

**Authors:** Ivan Phelan, Alicia Carrion-Plaza, Penny J Furness, Paul Dimitri

**Affiliations:** 1 Centre for Culture, Media and Society, College of Social Sciences and Arts, Sheffield Hallam University, Sheffield S1 1WB, UK; 2 Department of Psychology, Sociology and Politics, College of Social Sciences and Arts, Sheffield Hallam University, Sheffield S1 1WB, UK; 3 Sheffield Children’s NHS Foundation Trust, Sheffield Children’s, Sheffield S10 2TH, UK

**Keywords:** Virtual reality, Patient-centred design, Upper limb motor impairment, Pain management, Children’s rehabilitation

## Abstract

Upper limb motor impairment (ULMI) rehabilitation is a long-term, demanding and challenging process to recover motor functionality. Children and adolescents may be limited in daily life activities due to reduced functions such as decreased joint movement or muscle weakness. Home-based therapy with Immersive Virtual Reality can offer greater accessibility, delivery and early rehabilitation to significantly optimise functional outcomes and quality of life. This feasibility study aimed to explore the perceptions and impacts of an immersive and interactive VR scenario suitable for ULMI rehabilitation for children at home. It was analysed using mixed methods (quantitative and qualitative) and from a multidirectional perspective (patients, clinicians and family members). Amongst the main results, it was found that IVR for ULMI home rehabilitation (1) is easy to learn and acceptable; (2) improves motor function; (3) reduces the difficulty in the reproduction of therapeutic movements; (4) is motivating and enjoyable and (5) improves quality of life. This study is the first study on the use of IVR applied to home rehabilitation of ULMI in children. These results suggested that similar outcomes may be possible with self-directed IVR home rehabilitation compared to face to face conventional rehabilitation, which can be costly to both the patient and the healthcare system, decreasing the length of stay at the hospital and treatment duration. It has also presented an innovative solution to the Covid-19 emergency where children could not receive their clinic therapy. Further research is recommended to understand better the mechanisms involved in physiotherapeutic recovery and how IVR rehabilitation helps to improve conventional treatments.

## 1 Introduction

Paediatric Upper Limb Motor Impairments (ULMI) requiring rehabilitation affect a diverse population, including people affected by neurological, musculoskeletal and congenital conditions (e.g. cerebral palsy, fractures, scalds or brachial plexus birth injury) (National Institute for Health and Care Excellence 2017). Common problems in children and young people associated with these conditions include impaired joint movement, muscle weakness and decreased upper limb lean mass (Cambiaso-Daniel et al. 2017), leading to limitations in daily life activities due to reduced function and diminished quality of life (Liebs et al. 2020).

ULMI rehabilitation is a long-term, demanding and difficult process to recover motor functionality, which includes a conventional rehabilitation (CR) program consisting of stretching, strengthening, positioning, splinting, casting and facilitation of movement focussed on high-intensity, repetitive and task-specific practice (Pritchard-Wiart and Phelan 2018; Tarakci et al. 2020). Early rehabilitation contributes significantly to optimising functional outcomes and overall well-being to prevent acute problems from becoming chronic with potential cost savings (Neilson et al. 2019). Tackling non-pharmacological interventions to minimise procedural pain during physical therapies and treatment adherence is essential for successful long-term functional outcomes for this population (Threapleton et al. 2016). However, CR results in high costs to the healthcare system. Factors such as financial pressures on inpatient stay, geographic location and the number of therapists, especially in rural areas, can limit access to clinical therapy (Gueye et al. 2021). The functional declines observed after discharge from inpatient rehabilitation are undoubtedly exacerbated by this limited access to clinical treatment (Riddell et al. 2011). This problem has been compounded further by the COVID-19 pandemic, which has forced the suspension of outpatient physician visits due to containment measures, making it a challenge to provide continuum care for patients in the hospital (Goyal and Prawin 2021).

Home-based therapy could potentially overcome this problem, improving early functional recovery in musculo-skeletal impairments (Falvey et al. 2018) whilst limiting the need to travel for treatment. Recent studies have suggested that similar outcomes may be possible with self-directed home exercise programs compared to face to face CR (Wang, Rodon et al. 2019), decreasing length of stay at the hospital and treatment duration (Menon et al. 2020). Novel technology, such as Virtual Reality (VR), can offer greater accessibility and can be delivered remotely, cost-effectively and conveniently without transportation and provide individually tailored treatments (Su et al. 2021; Thielbar et al. 2020).

In the clinical setting, several trials have innovated and investigated effective treatments for ULMI recovery. They have identified VR technology as an opportunity to promote movement recovery during the physiotherapeutic process (Levac et al. 2013). VR-supported rehabilitation has a high intrinsic motivational power that increases engagement and enjoyment of therapeutic routines for adults and children (Campo-Prieto et al. 2021; Chen et al. 2014; Laver et al. 2017). This is very important because even if a proper rehabilitation therapy is prescribed, and it can fail because of the patient’s lack of motivation (López-Jaquero et al. 2019; Meyns et al. 2018). In addition, it is effective in reducing procedural pain, with minimal adverse effects and economical due to the reduced costs of the technology (Aydın and Özyazıcıoğlu 2019; Chan et al. 2020; Chen et al. 2020, Garrett et al. 2014; Walther-Larsen et al. 2019). It lets users immerse themselves in an engaging and challenging virtual scenario where interaction emulates the exercises required in their CR (Wenk et al. 2021). It also allows therapists to identify individuals’ difficulties and customise the control of variables, such as speed, size, distance and visual and auditory feedback (Brusque et al. 2018; Zahabi et al. 2020).

The “Impact VRLab” based in Sheffield has developed a suite of immersive and interactive VR rehabilitation systems for paediatric and adult patients with neurological and musculoskeletal conditions, orthopaedic trauma, and pain management (Chau et al. 2020; Furness et al. 2019; Phelan et al. 2019). Immersive scenarios possessing high degrees of the patient presence and engagement proved the most effective (Furness et al. 2019). A small scale feasibility study was carried out to help children with ULMI to perform their rehabilitation exercises in a clinical setting. Results showed a reduction in pain and difficulty ratings, increased enjoyment and improved range of motion (ROM). The objective measurement scales were captured using a goniometer measurement instrument to capture ROM (Phelan et al. 2021a, b).

This study aims to explore the feasibility, acceptability and perceived effectiveness of an overhauled Immersive Virtual Reality (IVR) system tailored for ULMI rehabilitation for children in their homes. Using feedback acquired through co-design with patients, caregivers and clinical staff, a new improved system specifically tailored for home use was developed.

Selected outcome measures include ROM readings, quality of life (PedsQL), usability (SUS) and qualitative data (interviews).

The redesign of the system builds on previous studies by incorporating additional immersive levels with new mechanics (arrow types) that replicate physiotherapy exercises (stretching, flexions and rotations). Usability required significant attention since the user would be using the system in their homes without instant support. This required the inclusion of a character that follows the user to provide tips on how to progress. Motivational challenges and engaging scenarios were implemented to maintain the users’ attention to prevent the therapy from failing, as is often the case with self-directed treatments.

An aim of this study is to provide rehabilitation to paediatric patients with ULMI to optimise functional outcomes and overall well-being and quality of life. Utilising IVR technology with self-directed home rehabilitation, this system attempts to decrease healthcare pressure due to the Covid-19 pandemic by reducing clinic visits. This alternative approach to rehabilitation at home does not require supervised sessions. Although the therapist still needs to perform an initial assessment along with training on how to use the system, the subsequent follow-up appointments should be reduced with the improvement in adherence.

## 2 Related work

VR scenarios can be presented through a head-mounted device (IVR), or a projection system or a flat-screen (non-IVR). The state of the art of clinical studies conducted to demonstrate the efficacy and acceptability of VR for ULMI rehabilitation has been primarily focussed on adults and non-immersive VR systems. There is a lack of clinical studies of IVR applied to children and in particular during home-based rehabilitation.

### 2.1 Non-IVR intervention

Non-IVR environments provide visual feedback, presented through projection systems or flat screens. Biofeedback enables control of the timing of the task and supports motor learning (Kitago and Krakauer 2013). The most frequently used devices are computer-based supported, such as Microsoft Kinect and Nintendo Wii-Fit (Kong et al. 2016; Simonsen et al. 2017).

#### 2.1.1 Intervention with adult patients: upper limb stroke impairments

The majority studies have been with adults with a focus on stroke patients. The randomised controlled trial (RCT) of Kiper et al. (2018) with stroke patients demonstrated the effectiveness of reinforced feedback in non-IVR treatment, combined with CR for upper limb and compared with just CR. Similarly, Lee et al. (2018) investigated the effects of game-based non-IVR canoe paddling training, when combined with CR programs, on postural balance and upper limb function in stroke patients (*n* = 30). Results showed significant improvements compared with the baseline measurements and significantly greater improvement in the experimental group compared with the control group (*p* < 0.05).

Shahmoradi et al. (2021) presented a single before–after study to evaluate the impact of the non-IVR games on UL rehabilitation in chronic stroke patients (*n* = 10; mean age 52). The games had positive effects on the shoulder abduction (16.26 ± 23.94, *p* < 0.02), adduction (59.24 ± 74.76, *p* < 0.00), wrist supination (0.06 ± 1.34, *p* < 0.03).

As one of the first studies to evaluate the use of VR for ULMI rehabilitation after stroke, combining non-IVR and home-based approaches, Piron et al. (2009) compared a remotely controlled programme to treat motor deficits in post-stroke patients *(n* = 36) with CR. The experimental treatment was a non-IVR virtual reality-based system delivered via the Internet, which provided motor tasks to the patients from a remote rehabilitation facility with the therapist’s support. Results showed the Fugl-Meyer Assessment Upper Extremity (FMA-UE) indicated a significantly higher improvement in post-treatment motor function in the experimental group.

Based on self-directed therapy, a different approach was presented by Wittman et al. (2016), who conducted an open-label, single-group study with stroke patients (*n* = 11). The patients trained with an inertial measurement unit (IMU)-based non-IVR system (ArmeoSenso) in their homes for six weeks. All subjects could use the system independently in their homes, and no safety issues were reported. The arm function of these patients improved significantly by 4.1 points (*p* < 0.003) in the FMA-UE. ArmeoSenso-based metrics showed an improvement in arm function, a high number of reaching movements (387 per session) and minimal compensatory activities of the trunk whilst training. Dodakian et al. (2017) designed and evaluated a home-based non-IVR rehabilitation program, Neurorehabil Neural Repair, for stroke patients (*n* = 12). Arm motor status showed significant gains (FMA-UE change 4.8 ± 3.8 points, *p* < 0.0015), with half of the participants exceeding the minimal clinically significant difference.

Similarly, to empower patients to control their treatment at home, Choi and Paik (2018) developed a mobile-based non-IVR program for UL stroke rehabilitation to promote patients’ engagement in rehabilitation therapy as a more exciting and motivating tool (*n* = 24). The system included a mobile device (tablet PC) for visualisation and a smart-phone to obtain information about the movement of the affected arm using the built-in sensor. Findings showed that the system effectively promoted UL recovery in patients with stroke.

All of the works included above use non-IVR systems, despite studies, suggesting that IVR using Head-Mounted Device (HMD) is more effective for upper limb motor recovery than non-IVR (Henderson et al. 2007).

#### 2.1.2 Interventions in paediatrics

Research with children has similarly been focussed on using non-IVR and has targeted children with cerebral palsy (Deutsch et al. 2008; Sandlund et al. 2009). Studies, such as those of Jannink et al. (2008), Sharan et al. (2021) and Sun (2012), reported a positive effect of using non-IVR game training on physical motor rehabilitation in children.

Recently, Tarakci et al. (2020) developed Leap Motion Controller-based training (LMCBT). They ran an RCT to investigate the potential efficacy of an 8-week LMCBT program set as a ULMI rehabilitation program by comparing CR in children and adolescents (*n* = 92) with physical disabilities such as juvenile idiopathic arthritis (JIA), cerebral palsy (CP) and brachial plexus birth injury. Comparisons between LMCBT and CR groups showed similar results in all parameters in all disease groups (*p* > 0.05). Thus, this study has quantitatively shown that LMCBT should be an effective alternative treatment option in children and adolescents with physical disabilities.

On the other hand, Choi et al. (2021) carried out an RCT to investigate the efficacy of a non-IVR rehabilitation system of wearable multi-inertial sensors to improve arm function in children with brain injury (*n* = 80). Results showed a significant improvement in upper limb dexterity functions (The Melbourne Assessment of Unilateral Upper Limb Function-2 (MA-2)), after treatment, significantly higher in the experimental group (DELTA = 10.09 ± 10.50) compare with the control group (DELTA = 3.65 ± 6.92) (*p* < 0.05).

Although the results found by these studies are relevant and demonstrate positive results, suggesting that non-IVR can help in the rehabilitation of children with ULMI, more research is needed for a broader range of clinical conditions and with systems with greater capacity for immersion and interaction.

### 2.2 IVR rehabilitation

IVR environments are presented to the patients through a HMD, and this technology increases the user’s level of immersion (presence) in the environment. This plays an essential role in providing an optimal condition for task practice, as does the meaning of the task for the user (Kitago and Krakauer 2013; Martirosov et al. 2021). In the literature, we found studies in which IVR was used to improve UL rehabilitation. This mainly focussed on stroke and CP injuries, where paediatric upper limb injuries require a task-specific physiotherapeutic approach different from injuries and burns (Bortone et al. 2020; Mekbib et al. 2020, 2021). Interestingly, only one study was conducted with the paediatric population.

Mekbib et al. (2020) designed and then, implemented a neuroscience-based IVR protocol to rehabilitate stroke adult patients. The system provides unilateral and bilateral limb reflex exercises in a fully immersive virtual environment that can stimulate and activate the mirror neuron system in the brain to assist rehabilitation in subacute stroke patients (*n* = 12). Following the intervention, the primary outcome was that patients demonstrated a significant improvement in their motor function FMA-UE scores (*p* < 0.042) used to quantify the motor recovery status. One year later, (Mekbib et al. 2021) conducted an RCT of the same designed system. Twenty-three participants were randomly assigned to a VR group (*n* = 23, IVR-group *n* = 12; control group *n* = 11). Both groups significantly improved the Barthel Index (BI) (*p* < 0.05), reflecting the recovery of UL motor function. The IVR-group revealed more significant improvements in FMA-UE scores (*p* < 0.05) than in the control group. Neural activity increased after the intervention, particularly in brain areas involved in the recovery of motor functioning, such as the primary motor cortex.

The only study with children was carried out by Bortone et al. (2020) and aimed to determine the efficacy of immersive Virtual Environments and weaRable hAptic devices (VERA) for rehabilitation of ULMI in children (*n* = 8) with CP and developmental dyspraxia (DD). A two-period cross-over design was adopted for determining the differences between the proposed therapy and CR. Eight children were randomised into two groups: one group received the VERA treatment in the first period and the manual therapy in the second period and vice-versa for the other group. Results show both groups significantly improved their performance. No statistically significant differences were found between the two groups. The study suggested that IVR and wearable haptic devices are a viable alternative to CR for improving upper limb function in children with neuromotor impairments.

This review of related works has allowed us to conclude that VR can be used to improve ULMI rehabilitation, but that not much is known about the impact in the paediatric population, and that no studies have evaluated the feasibility, acceptability and perceived effectiveness of using an IVR system in particular for home rehabilitation and with children.

## 3 Methods

### 3.1 Participants

Ethical approvals were obtained from NHS Health Research Authority (London—Bloomsbury Research Ethics Committee (IRAS Ref. 274,257)) and Sheffield Hallam University Ethics Review Committee (Ref. ER19710920). Permissions were also gained from local NHS Hospital Trust Research and Development (R&D) (Ref. SCH-2540). We recruited participants aged 7–16 from Sheffield Children’s NHS Foundation Trust (FT), who had sustained ULMI requiring rehabilitative care. Exclusion criteria included: 1) injuries to the face or head that could hinder the correct positioning of the HMD or pose an infection risk; 2) a learning impairment that could hinder the understanding of the task; 3) a history of severe motion sickness; 4) mental health problems and 5) inability to speak and understand English. Parents of eligible children, present during the rehabilitation treatment, were included in the study to provide feedback on their children’s experience with VR game and their perspective on using VR.

Considering these criteria, eight patients, three boys and five girls, ages 7 to 16 years (M = 13.25, SD = 2.91) and their eight mothers, participated in this study. Patients (Pt) had various UL injuries (summarised in Table 1).

An OT from the Sheffield Children’s NHS FT for Burns, Plastics and Orthopaedics recruited the patients, gave out devices, administered the outcome measures and provided us with feedback about their experience with the VR game.

### 3.2 Equipment: IVR system

Equipment included an IVR Meta Quest HMD with touch controllers. The development software comprised Unreal Engine 4.23, 3ds Max 2021 and Substance Designer 11.3. The Meta Quest performance was a limiting factor that required the system to be completely rebuilt. Interactive scenarios combining elements of archery and climbing that were suitable and customisable for children requiring upper limb rehabilitation were developed by Impact VRLab in consultation with physiotherapists and piloted with school children before the clinical trial. In a previous clinical trial (Phelan et al. 2021a, b), the game setting, characters and interactive elements were shown to be engaging and a feasible approach to help children with ULMI rehabilitation in a clinical setting. However, the designer developed some key aspects to make the game more engaging and effective. Therefore, for this study, we used an new enhanced version that includes:

#### A narrative and tutorial

**Fig. 1:**
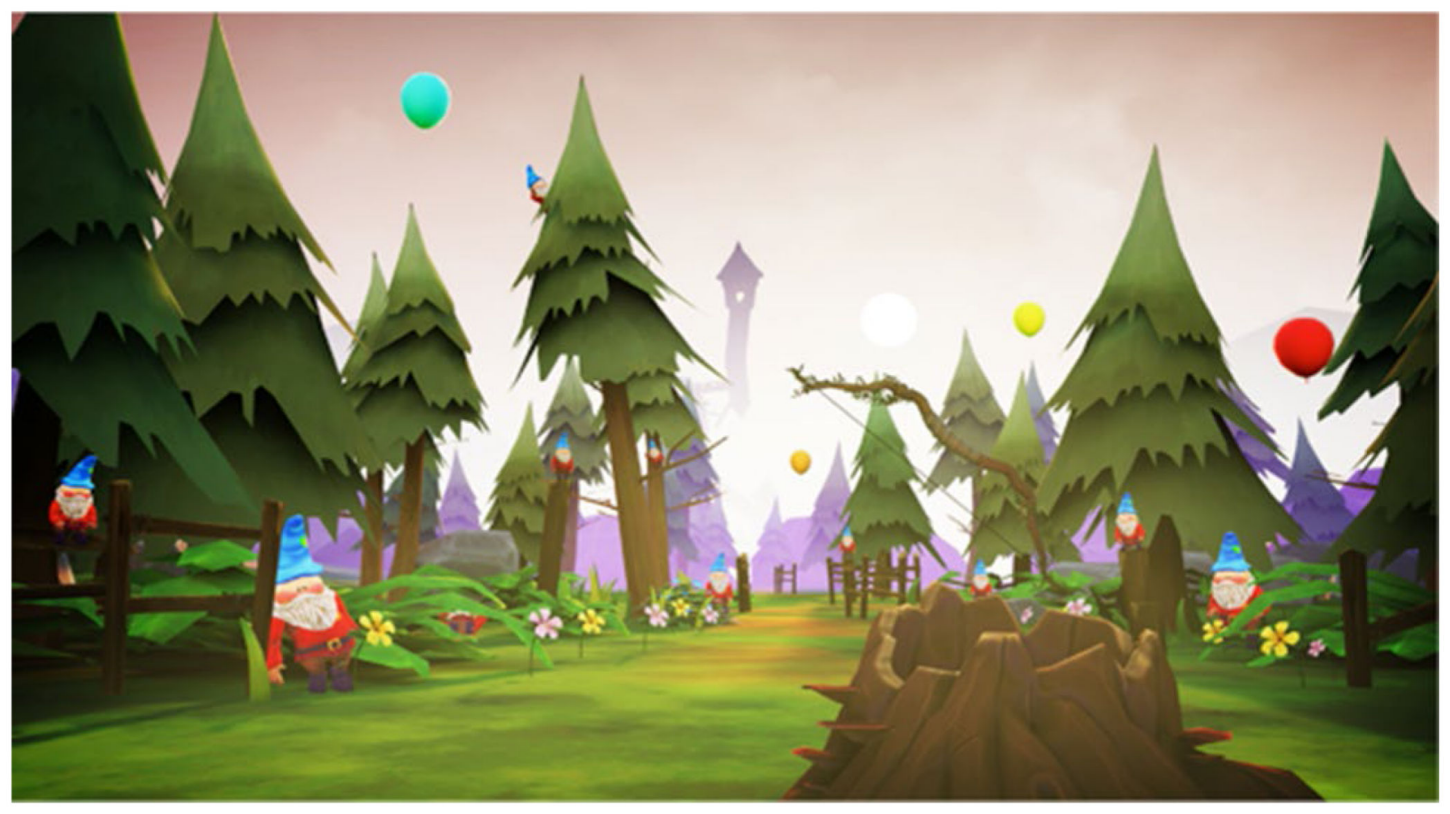
New scenarios: Forest level. A woodland environment with balloon targets and destructible gnomes acted as a tutorial for the archery mechanic

**Fig. 2:**
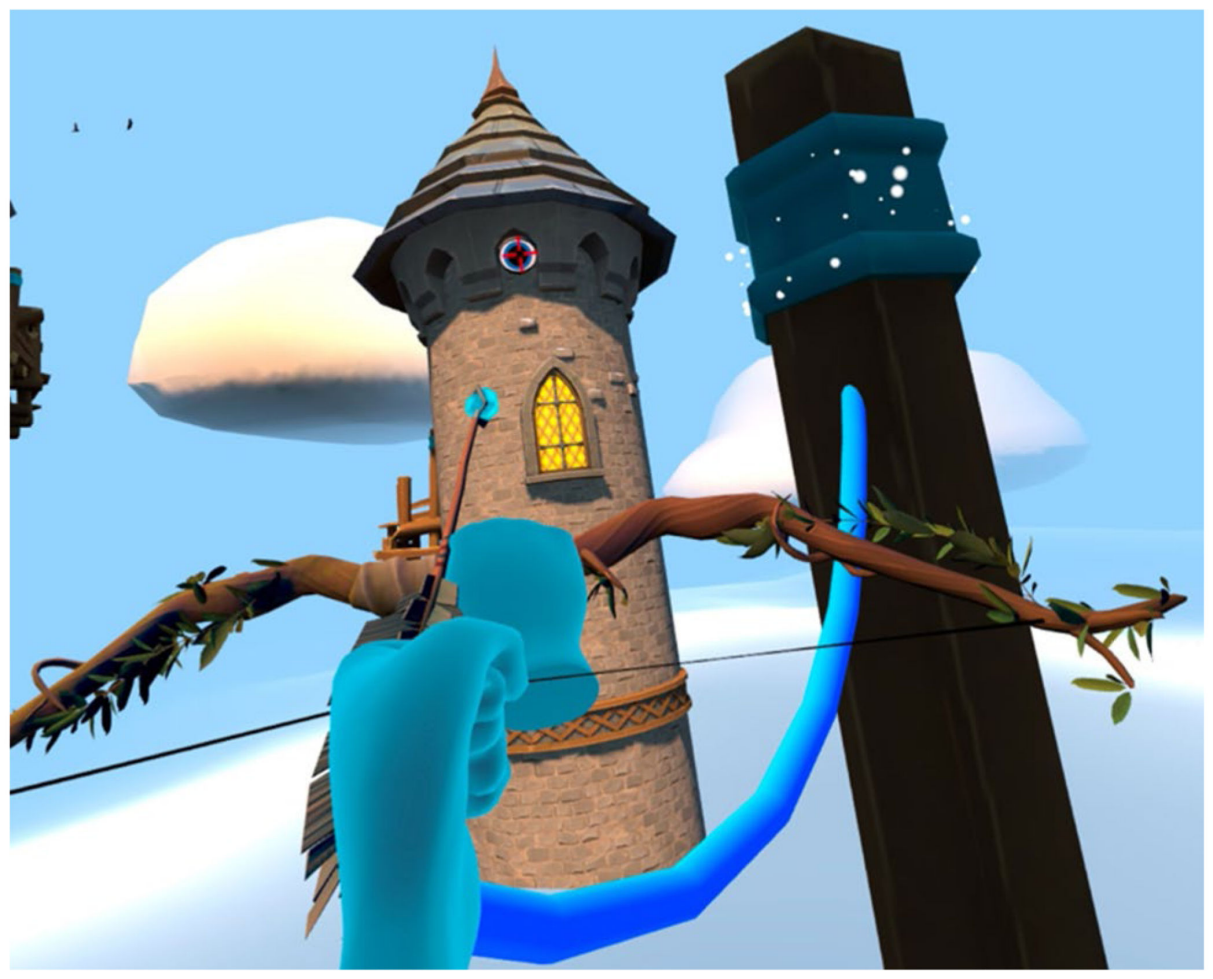
New scenarios: Tower level. An attached rope (rope arrow) could be tethered to two points to create a rope bridge. An exciting space with more intricate puzzles

**Fig. 3:**
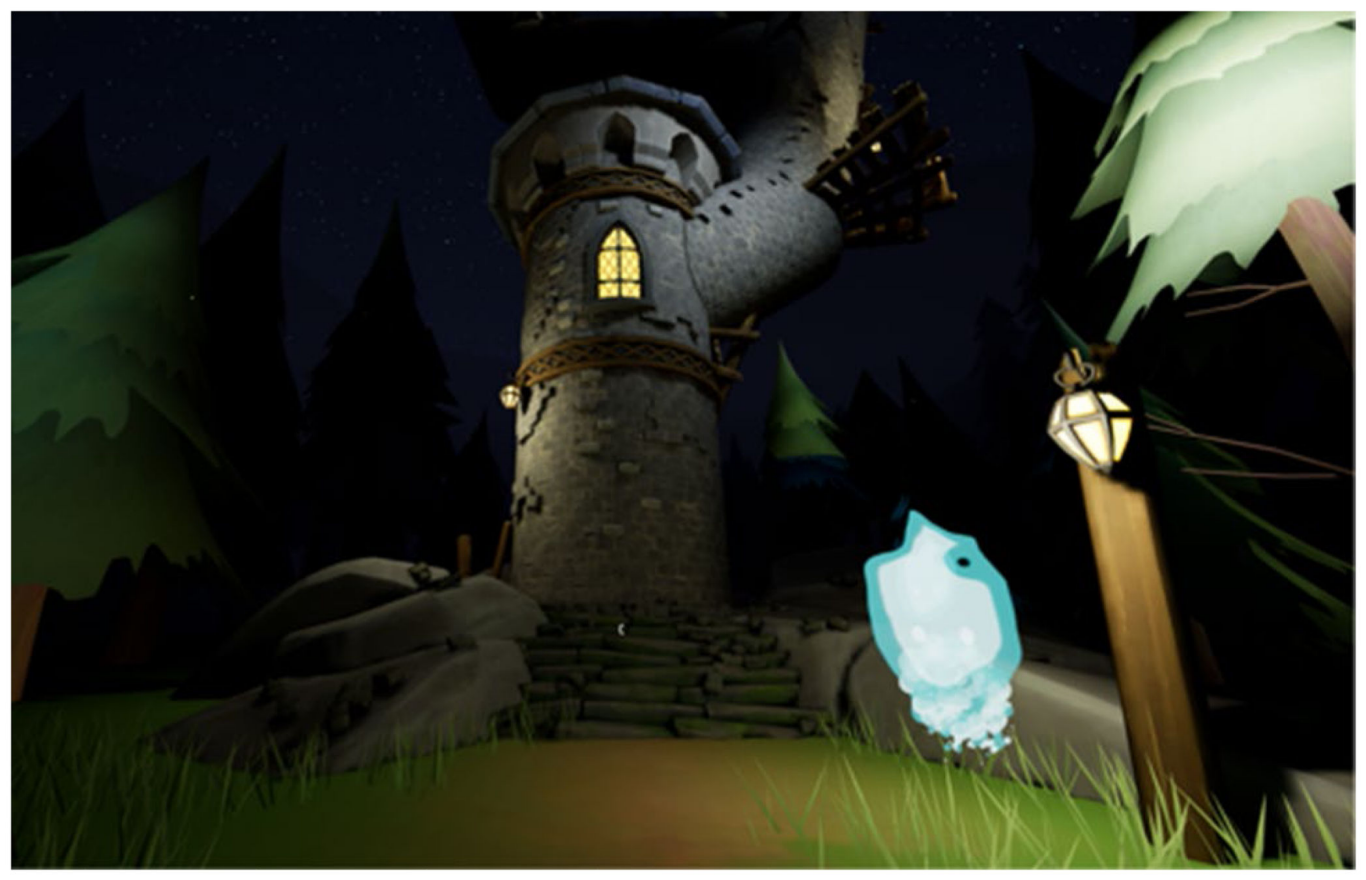
The companion. It is an animated blue flame that follows the player giving clues and highlighting points of interest

The narrative is partly told through the environment. Objects in the world are presented to suggest a story to the user. Each level introduced a new gameplay mechanic and visual identity. For example, the Forest level (Fig. 1) acted as a tutorial for the archery mechanic, followed by the Tower level (Fig. 2) to introduce climbing. A mechanic was devised by using an arrow with an attached rope (rope arrow) that could be tethered to two points to create a rope bridge. This gave the user more freedom to traverse the area, gaining a sense of autonomy whilst still requiring arm movements. A companion (Fig. 3) was introduced that followed the player providing hints and highlighting points of interest that helped deliver the narrative and create a more believable and engaging world.

#### New climbing and archery levels

**Fig. 4:**
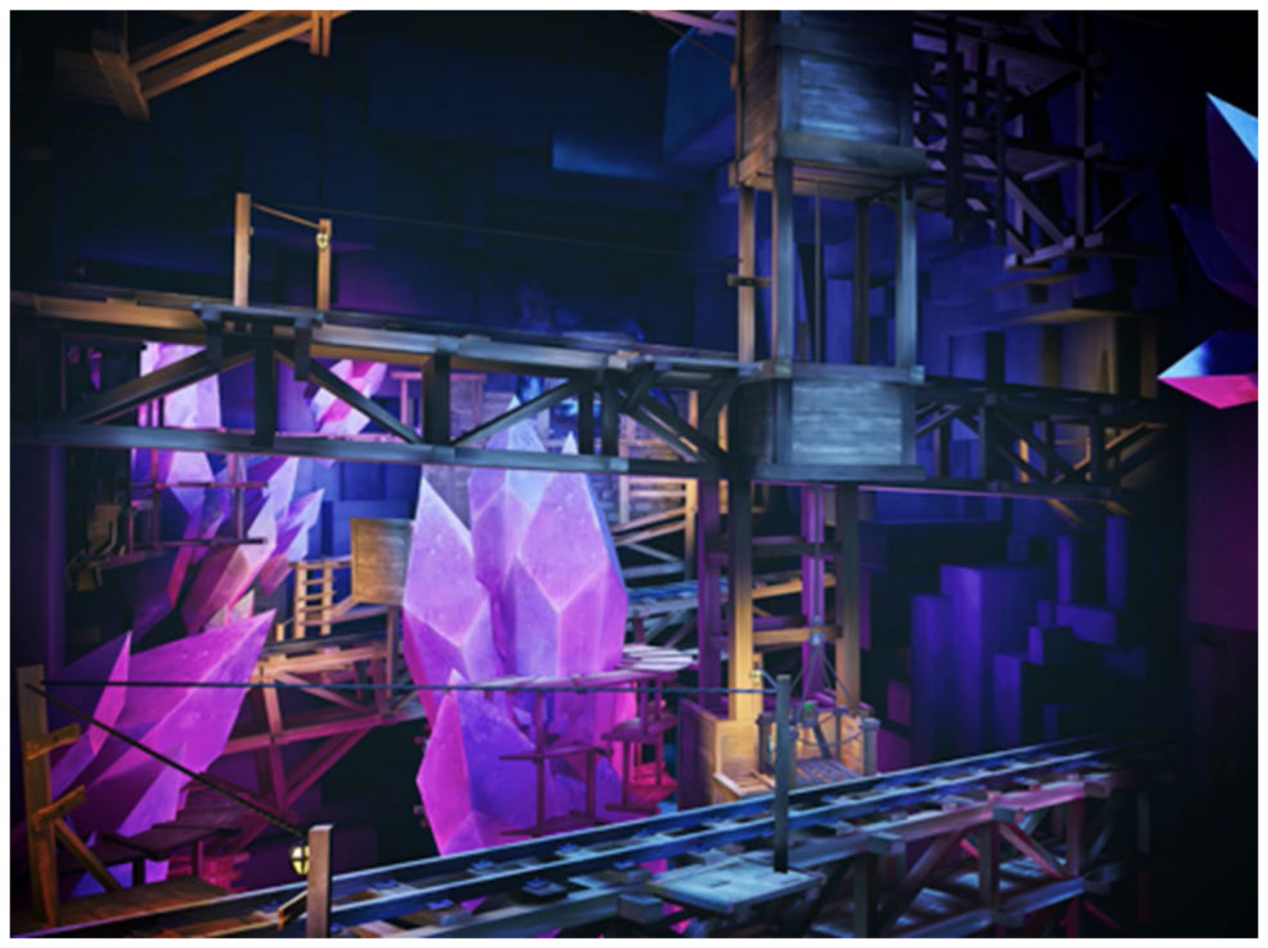
New scenarios: Mine level. A fantasy diamond mine with platforms, levers and carts that the player has to interact with. The teleport arrow mechanic is introduced along with the other arrows. This provided a challenge to the user and a chance to test out their skills

More game levels have been included to allow children to progress through the game’s challenges and achieve a more complex gaming experience. The sky Tower level (Fig. 2) utilised the rope arrow to produce an exciting space with more intricate puzzles. The Mine level (Fig. 4) used the teleport arrow mechanic along with all the other arrows. This provided a challenge to the user and a chance to test out their skills.

#### New elements

Hidden collectable items, recorded scores, bows unlockable and trophies were included as incentives for the player. These features encourage the user to get them out and replay the levels to get 100% completion.

## 3.3 Instruments

### A goniometer (Standard BASELINE® 12-inch)

was used by the physiotherapist to measure a Range of Movement (ROM) of the affected and unaffected upper limb join (flexion, extension, abduction and adduction) before and after the at-home trial. This offered the opportunity to compare usual movement without an injury and the IVR rehabilitation.

### Paediatric Quality of Life Inventory (Version 4.0 – UK English) (PedsQL)

is a standardised assessment for children aged 5–18 and was completed by patients before and after the IVR rehabilitation at-home trial. The scale features twenty-three items representing five dimensions: Physical, Emotional, Social and School Functioning (e.g. physical functioning (problems with…) walking 100 m; emotional functioning (problems with…) feeling afraid or scared; social functioning (problems with…) getting along with other children; and school functioning (problems with…) paying attention in class). The instructions ask how much of a problem each item has been in the past month, and responses are on a five-point scale ranging from 0 (never a problem) to 4 (almost always a problem) (Upton et al. 2005). Items on the PedsQL are reverse scored and transformed to a 0–100 scale. Higher scores indicate better health-related quality of life: 0 (“Never”) = 100; 1 (“Almost Never”) = 75; 2 (“Sometimes”) = 50; 3 (“Often”) = 25 and 4 (“Almost Always”) = 0. To create the Psychosocial Health Summary Score, the mean is computed as the sum of the items over the number of items answered in the Emotional, Social and School Functioning Scales. The Physical Health Summary Score is the same as the Physical Functioning Scale Score. Internal reliability exceeded 0.70 for all proxy and self-report sub-scales.

### System Usability Scale (SUS)

is a quantitative scale to measure system usability completed by patients after the IVR rehabilitation at-home trial. It consists of 10 items with 5-point response options from Strongly agree to Strongly disagree (Brooke 1996). The scale evaluates the perceived ease of use (a single dimension). However, recent research shows that items 4 and 10 could provide the learnability dimension (Lewis and Sauro 2009). Score contribution of each item was coded from 0 to 4 (items with positive words 1, 3, 5, 7 and 9; items with negative words 2, 4, 6, 8 and 10), and the sum of the contributions of the item scores was multiplied by 2.5 to obtain the overall SUS score (0 to 100). Scores were analysed above or below the standard mean score of 68. A score close to 100 was considered good usability of the system.

### Semi-structured interviews

were conducted by the research nurse in person (Pt 1, 4, 6, 7) and by phone (Pt 2, 3, 8) with patients and parents after the IVR rehabilitation at-home trial. Four closed ten-point Likert questions provided a quantitative measure of the perceived ease or difficulty of use, pain levels and enjoyability of the IVR system. Open-ended questions provided qualitative data relating to difficulty, pain and enjoyability and participant attitudes towards the IVR system and its future deployment. Another semi-structured interview was conducted with the OT by the research nurse in person at the end of the trial to explore her attitudes towards the IVR system and future VR deployment. Both interviews were recorded, transcribed and anonymised.

## 3.4 Procedure

The OT was trained by a member of the research team to know how to use the IVR games and to be who recruited the patients, collected informed consent, gave out devices and administered the outcome measures.

Before consenting to participate, each patient was offered a 5–10 min experience of the game to familiarise themselves with how it worked and to check for problems. If they agreed with the participation, written and verbal consent were taken from parents and children. After this, patients had, as in CR, a 20–30 min appointment in which the OT reviewed their previously prescribed home exercise regime with them, measured ROM and pre-trial PedsQL and explained and discussed new home exercises. For the at-home trial, patients were asked to use the IVR games to do their prescribed home exercises. Children were then asked to use the IVR system for approximately 15 min twice a day at home for three weeks.

Following the at-home trial, patients and parents returned the equipment to the clinic in the final appointment, and the SUS and the post-trial PedsQL were administrated to the patient by OT in person. In addition, they participated in semi-structured interviews.

At the end of the trial, an interview (10 min) with the OT was conducted by a research nurse.

## 3.5 Analysis

Quantitative data (ROMs readings, PedsQL, SUS scores and Likert questions) were analysed and reported by descriptive analysis (median and range statistics) and means comparison (nonparametric: Wilcoxon signed-rank test (Wilcoxon 1945)) pre- and post-IVR rehabilitation trial using the statistical package SPSS v.26 (Field 2009). Qualitative data from the interviews were analysed using inductive, semantic content analysis (Hsieh and Shannon 2005) by two qualitative analysts.

## 4 Outcomes

The quantitative and qualitative analysis results will be presented in this section.

### 4.1 Goniometer: range of motion results

The OT measured the ROM of the affected upper limb joint before and after exposure to IVR (5–10 min) and the unaffected upper limb to have a comparative value of the usual movement without an injury. As shown in Table 2, most, though not all, of the affected area ROM readings showed improvements in movement post-VR therapy. The remaining readings indicated unchanged movement (Pt 5 wrist pronation, radial and ulna deviation, patient 6 wrist radial and ulna deviation and Pt 8 shoulder external and internal rotation).

Compared to healthy limb ROM measured at the same joint area, five of the eight patients achieved post-VR measures equal to the usual degrees of motion at some joint assessed (Table 2).

### 4.2 Paediatric quality of life inventory results

PedsQL results are shown in Tables 3 and 4. On each dimension and as an overall measure, post-VR scores showed statistically significant improvements (*p* ≤ 0.05).

### 4.3 System usability scale results

The direct item scores were transformed, summed and converted to 0–100. The mean obtained by the patients was 99.64 (*n* = 7), demonstrating high usability above the standard mean score of 68. In addition, patients strongly agreed with items such as “I think I would like to use the VR system frequently”, “I found the VR system easy to use” and “I felt very confident using the VR system”.

Items 4 and 10, referred to as the learnability dimension, received a direct score (0–4) of 4 and 3.43, respectively, showing that the VR system was easy to learn. In addition, patients reported not needing technical assistance before using the system.

### 4.4 Interview results

Quantitative data from the Likert questions showed that, in general, patients reported no difficulties in setting up and using the game, nor did they perceive pain when using it, with high levels of enjoyment as shown in Table 5 (*M* = 9.29, *ST* = 0.951; *M* = 3.17, *ST* = 1.60; *M* = 1.33, *ST* = 1.75: *M* = 9.00, *ST* = 1.29).

Qualitative comments from all 15 participants (7 patients, 7 parents and 1 OT) demonstrated positive impressions of and positive attitudes towards IVR during ULMI rehabilitation (Table 6). Parents (6/7) found the IVR game an excellent idea that keeps them doing the necessary physio exercises. They also thought it would have a good place in the clinic in the future, not just for ULMI rehabilitation but also for other pathologies. The OT also considered that the IVR system could be helpful in different areas. She thought that it would be asked to be used again by patients and parents, those who already tried it, and others new ones that could come to the service.

**Fig. 5:**
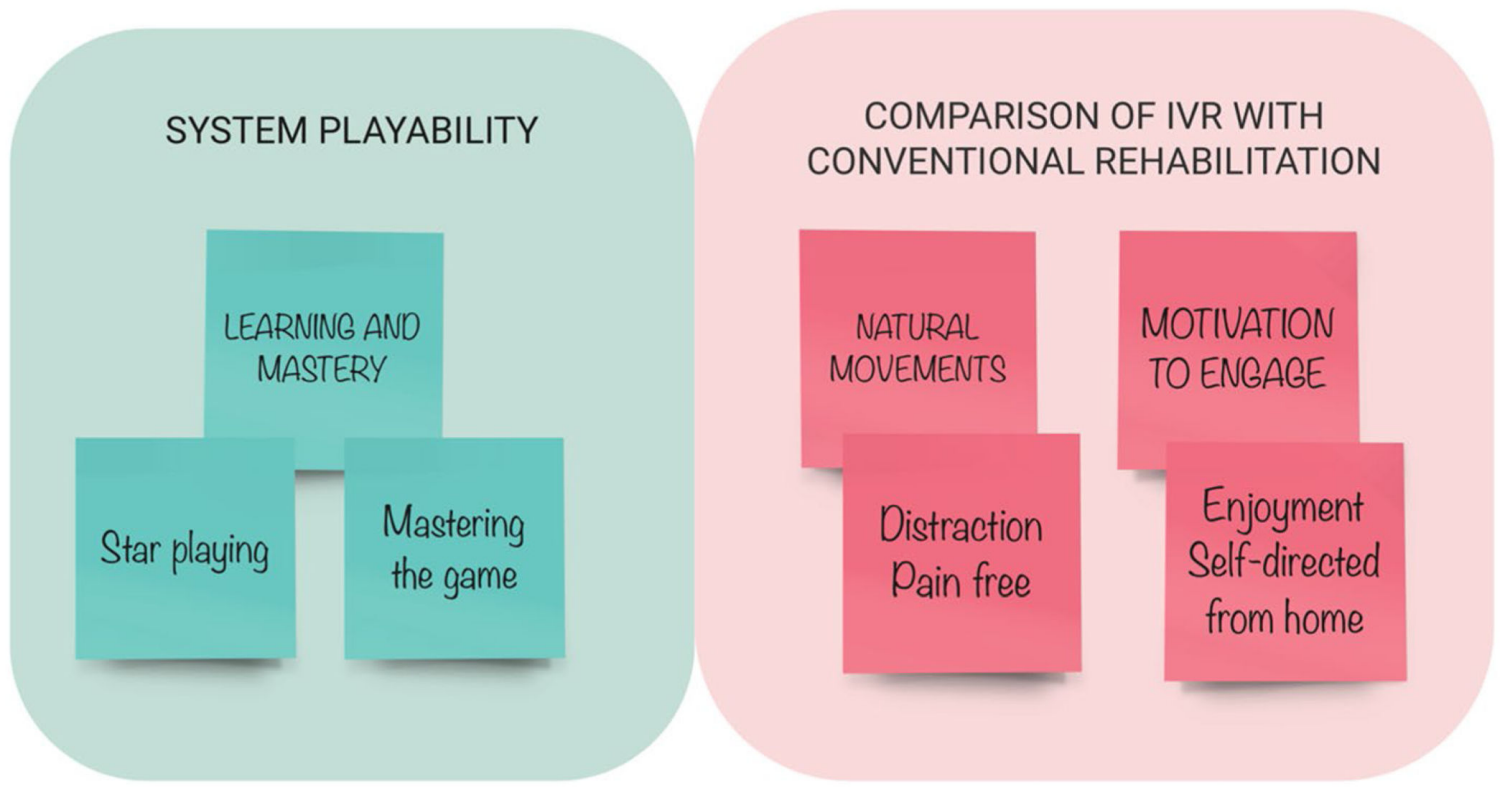
Content analysis themes from the patients, parents and OT interviews: “System Playability” and “Comparison of IVR with CR”

The content analysis generated two main themes. One theme related to “System Playability” was characterised by a process of “Learning and Mastery”. The second theme focussed on “Comparison of IVR with CR” and included sub-themes of: “Natural movements” and “Motivation to engage” (Fig. 5). Data extracts can be found in Tables 7, 8 and 9.

#### 4.4.1 Natural movements

IVR rehabilitation aims to facilitate the specific movements recommended by OTs in CR. In this study, the OT stated that this IVR intervention successfully reproduced the required exercises and combined all the movements she was looking for.

Parents (5/7) observed that when their child was immersed in the game, he performed movements more fluently and naturally, without realising it. Similarly, most patients (4/7) and parents (6/7) indicated that the game made rehabilitation exercises easier to complete. This ease seemed to reflect both fluency of movement and lack of pain and fear. Reported pain levels were low (*M* = 1.33, *ST* = 1.75). Qualitative data from parents suggested that they (6/7) did not witness pain in their children and perceived that children were not conscious of doing the rehabilitation exercises. Parent 8 reported that IVR reduced the fear and limitation of movement in her child with an external fixator, thereby helping her move naturally and unconsciously. As a result, she realised that she could do more than she had previously thought and achieved a better range of motion.

#### 4.4.2 Motivation to engage

Patients (5/7) believed they would be more likely to complete their rehabilitation exercises with the IVR game at home. The IVR positively influenced their motivation and intention to carry on with the rehabilitation (6/7). Four noted that IVR rehabilitation was more fun and less boring, and quantitative data demonstrated high levels of enjoyment amongst participants (*M* = 9, *ST* = 1.30).

Linked to motivation, the frequency of use was high. Three out of seven patients used it every day for between 10 and 20 min. One patient stated that they used it at least twice a day. In only one case, a participant (Pt 4) reported reducing the frequency of use after the first week due to repeatedly playing the same games. The greater motivational effects of IVR resulted in some parents (3/7) feeling less that they needed to remind and motivate their child with exercises. OT perceptions supported these findings that patients found the IVR game more interesting, engaging and motivating than usual exercises and that movement had improved.

The best bits of the game reported by the patients were: shooting the arrows at the gnomes; seeing the score improve; rock climbing and falling; feeling challenged; being able to do different activities; graphics and animation. They also enjoyed being present in a virtual environment that they had never experienced previously. There were no worst bits reported from most patients (5/7). However, one patient (1/7) expressed that the worst was to have to do the levels before you can get onto the climbing one (Pt 2), and another (1/7) said that there are not that many games on it (Pt 4).

Data extracts are found in Table 9.

## 5 Discussion, limitations and future line of study

Children and adolescents with ULMI often undergo a long-term, demanding and challenging rehabilitation process to recover motor functionality (Liebs et al. 2020). Home-based therapy with VR has been shown to offer greater accessibility, delivery and early rehabilitation to improve functional outcomes and quality of life (Su et al. 2021; Thielbar et al. 2020). This study aimed to explore whether IVR could offer a feasible solution for the rehabilitation of children with ULMI at home. Using quantitative and qualitative approaches, we found that IVR has clear potential to be a successful solution for physical rehabilitation for children with ULMI. More importantly, our findings suggested that IVR appeared to be very effective as a self-directed home therapy for this clinical population. In summary, our results indicated that IVR for ULMI home rehabilitation could: (1) be easy to learn and acceptable, (2) improve motor function, (3) reduce the difficulty in the reproduction of therapeutic movements, (4) be motivating and enjoyable and (5) improve quality of life.

The ROM (Goniometer) data suggested that IVR rehabilitation could improve shoulder, wrist and elbow motor functionality, enhancing flexion, extension and abduction movements. The patients demonstrated ROM gains, in some cases even recovering the same degree of movement in the injured limb as the unaffected limb. These data are promising, indicating that all paediatric patients who have used our IVR game have improved their affected limb’s range of motion.

Quality of life (PedsQL) scores also indicated a significant improvement after the IVR home trial compared to baseline scores. Physical and psychosocial health scores improved after the study, which may reduce the risk of long-term diseases such as chronic pain and disability (Cambiaso-Daniel et al. 2017; Liebs et al. 2020).

The usability test (SUS) scores showed that patients generally understood the game mechanics well, and there were no critical errors that would have impeded the gameplay. For example, based on the patient’s subjective report, no difficulty was found in setting up and getting used to the game. In this sense, we must point out the relevance of having the OT at the initial session, where they were shown how to use the game. Therefore, we encourage researchers and clinicians when proposing a self-directed session for children at home to include a first contact with the game accompanied by the OT or other healthcare professionals delivering the therapy. In addition, we recommend offering follow-up or support via telephone or email to solve potential problems during home ULMI rehabilitation.

Furthermore, the positive results in learning and mastering the game may be explained by the inclusion of a tutorial at the beginning of the game, based on the findings of our previous study (Phelan et al. 2021a, b). As a result, children learned to reproduce the basic movements, and interaction mechanics were adapted to their capabilities, such as achievable range of movement. This is in line with current research, which demands games that allow the customisation of game variables to the individual’s difficulties (Brusque et al. 2018; Zahabi, Mohammed, and Razak 2020).

Another key element that helped avoid patient confusion was the inclusion of a companion that guides the player and acts as a narrator of the story. The companion was effective in the delivery of the narrative to create a more believable and engaging world. Also, consistent visual cues were added to interactable objects, and audio cues were incorporated to direct the player’s attention.

On the other hand, the qualitative results have contributed to detecting some IVR system usability improvement opportunities. Younger children (7–12 years old) reported they found it harder to get used to the game, whilst adolescents (aged 15–16) would like additional levels. This underpins future challenges in the development of IVR systems to support therapy across a broad age range of children. We recognise the need to maintain a certain difficulty level whilst being stimulating and providing meaningful activities during physical rehabilitation to ensure compliance, especially for children. There is scope for further development to ensure the right level of challenge and mastery for children of all ages. We plan to offer a broader range of difficulties in future iterations to ensure the enjoyment of those children who find it more difficult (e.g. younger patients in the early recovery phase or with complicated clinical conditions). In addition, this could positively affect self-confidence, self-esteem, attention span, concentration and interest in learning and, thereby, help to maintain motivation and compliance, as noted by previous researchers (Meyns et al. 2018; Wenk et al. 2021).

The OT reported that getting used to the game with the younger patients was challenging. As a result, the OT requested a way for the professional to monitor the system in real-time, observing what patients are seeing to better help and guide them in cases where they are younger or if they cannot make progress. Future development will consider this aspect.

Individualising the users’ exposure could also make the intervention suitable for people who experience side effects of motion sickness and dizziness. In our study, one patient reported feeling dizzy and believed this was due to playing for a long time. Customising the users’ exposure could involve the therapist setting safe playing times to limit these effects so that the system reminds the player to rest in cases where it is more at risk, such as those who suffer motion sickness (Martirosov et al. 2021).

Another finding from the combined OT, parent and patient data is that the game successfully reproduced the movements required by the physical rehabilitation therapy and prompted more movement CR. In addition, the game mechanics included many natural interactions, such as grasping, dragging or stretching, to smoothly and precisely replicate the rehabilitation movements safely (Wenk et al. 2021). Therefore, a co-design approach involving designers and physiotherapists and an iterative design process could be an essential aspect of designing this kind of system (Phelan et al. 2021a, b).

The qualitative data from the patients, parents and healthcare professionals highlighted that ULMI-IVR rehabilitation therapy for paediatric patients effectively improved motivation and maintained patients’ interest in performing the exercises from home. Furthermore, both the patients and their parents agreed that having the IVR game available increased the likelihood of achieving therapy at home. In our study, this is one of the most salient findings, because although self-directed home-based rehabilitation therapies have many benefits (early rehabilitation, early discharge from a hospital, reduced therapy time and being cost-effective), and they can fail because of the patient’s lack of motivation affecting therapy (Garrett et al. 2014; López-Jaquero, Montero, and Teruel 2019; Piron et al. 2009; Su et al. 2021; Thielbar et al. 2020; Wittmann et al. 2016). Home-based therapy can burden parents with the responsibility of overseeing children’s rehabilitation exercises, especially if children lack motivation. In this study, IVR seemed to reduce this burden: parents did not have to remind their children, and the children were proactive in wanting to exercise with the IVR. It is essential that the children feel motivated and enjoy their activity, as this rehabilitation can be long, repetitive and perceived as painful. Increasing therapeutic adherence is vital to achieving recovery of motor function and avoiding long-term problems.

In support of these findings, children reported that the most satisfying parts of the game were the more active parts of shooting gnomes and climbing. This result is in line with our previous study, where it was found that active scenarios were perceived as more engaging, challenging, distracting and immersive than passive ones reducing subjective awareness of pain (Phelan et al. 2019). Also, they highly valued being immersed in a virtual environment that they had never experienced before. This result would support the use of IVR over non-IVR for effective ULMI recovery (Henderson et al. 2007). Finally, patients reported that they enjoyed seeing how their scores increased. This leads us to believe that this competitive mechanic motivates them to continue their rehabilitation whilst enjoying it (Campo-Prieto et al. 2021).

This study has some limitations that should be taken into account to interpret the results. Firstly, this study lacks a control group and has a small sample size. However, given that the participants all had previously undergone conventional rehabilitation (CR), this allowed a comparison of CR to IVR rehabilitation in the qualitative feedback. Additionally, the VR system was used to treat a variety of ULMI, which allowed us to look at different aspects of interaction design and deployment considerations. Finally, using mixed methods (quantitative and qualitative) and from a multidirectional perspective (patients, clinicians and family members) improved the breadth and depth of understanding of IVR acceptability.

Future studies should examine IVR for clinical rehabilitation in a large-scale sample comparing the effectiveness of IVR rehabilitation with CR. In addition, to determine whether self-directed IVR home rehabilitation can be costly for both the patient and the healthcare system (Wang et al. 2019) and decrease the length of hospital stay and duration of treatment (Menon et al. 2020), we will conduct an economic and impact analysis that will help us determine the cost savings aligned with IVR home therapy and associated benefits (Neilson et al. 2019).

Future research could further assist practitioners deliver their interventions by developing scenarios with mechanics which facilitate movements as yet unaddressed by the current system. A reporting system for the therapist to assess the patients progress and usage could provide staff with valuable information to determine the stage of healing and if the difficulty should be altered. Increasing customisation options to provide training tailored to the patient’s abilities could help maintain attention. Adding more variety and valuable exercises for daily activities that are fun and challenging can increase motivation and adherence to treatment (Gerber et al. 2016; Kiper et al. 2018).

To summarise, this is the first feasibility study to trial IVR in the home rehabilitation of ULMI in children. The findings demonstrate that an IVR system has been developed capable of delivering ULMI therapy that leads to an increase in limb function, which is supported by the ease of use, enjoyment, adherence to the treatment, and can be delivered at home. It has also presented an innovative solution to the Covid-19 emergency where children could not receive their therapy in the hospital.

## Figures and Tables

**Table 1:** Patients ID and demographic data

ID	Injury	Age	Gender
Pt1	Back and arm (dog bite injury)	12	Female
Pt 2	Back and arm (scald)	7	Female
Pt 3	Wrist (assault fracture)	16	Female
Pt 4	Shoulders and upper arms (scald)	14	Male
Pt 5	Humeral and nerve (fracture)	15	Male
Pt 6	Arm and nerve (stab weapon injury)	15	Male
Pt 7	Elbow (arthrotomy operation)	15	Female
Pt 8	Arm (congenital disease, external frame)	12	Female

**Table 2:** Descriptive pre-VR, post-VR and unaffected ROM data

Patient ID	UL affected	Joint movement	ROM (degrees)		
			Pre	Post	Unaffected limb
Pt 1	Shoulder	Flexion	159	170*	175
		Extension	52	55**	55
		Abduction	112	165*	170
		External rotation	81	90**	90
		Internal rotation	52	85**	85
Pt 2	Shoulder	Flexion	168	174*	178
		Extension	51	55**	55
		Abduction	170	175**	175
		Internal rotation	69	75**	75
Pt 3	Wrist	Flexion	25	54*	78
		Extension	35	56*	83
		Supination	75	85*	90
		Pronation	80	85*	90
		Radial deviation	7	15**	15
		Ulna deviation	12	30**	30
Pt 4	Shoulder	Flexion	168	172*	180
		Extension	57	60*	65
		External rotation	82	90**	90
		Internal rotation	74	86*	90
Pt 5	Elbow	Flexion	118	120*	135
	Wrist	Flexion	75	80*	85
		Extension	80	82*	85
		Supination	83	85*	90
		Pronation	80	80	90
		Radial deviation	20	20	30
		Ulnar deviation	10	10	15
Pt 6	Elbow	Flexion	90	130**	130
	Wrist	Flexion	50	58*	85
		Extension	20	24*	85
		Pronation	80	85*	90
		Radial deviation	10	10	15
		Ulnar deviation	10	10	30
Pt 7	Elbow	Flexion	109	122*	140
Pt 8	Shoulder	Flexion	170	175*	180
		Extension	50	60*	65
		External rotation	55	55	80
		Internal rotation	55	55	80
	Wrist	Flexion	35	40*	80
		Extension	62	65*	80
		Supination	75	80*	90
		Pronation	75	80*	90
		Radial deviation	5	10*	15
		Ulnar deviation	23	25*	30

*ROM* Range of Motion in a joint, based on goniometer readings, *UL* Upper limb

* Indicates an increase in post-IVR ROM measured in comparison with the pre-IVR

** Indicates an increase that has come to match the movement of the unaffected limb at the same ROM movement evaluated

**Table 3:** PedsQL pre-post: Physical Health Summary

Patient ID	PF		
	Pre	Post	%
Pt 1	68.80	90.60	31.69
Pt 2	56.30	81.30	44.40
Pt 3	43.80	62.50	42.69
Pt 4	78.10	81.30	4.10
Pt 5	43.80	65.60	49.77
Pt 6	40.60	53.10	30.79
Pt 7	68.80	81.30	18.17
Pt 8	37.50	65.60	74.93
All	55.1	72.7*	31.94

*PF* Physical functioning scale score (items: PF1 = Walking 100 m; PF2 = Running; PF3 = Participating in sports; PF4=Lifting something heavy; PF5 = Taking a bath/shower; PF6 = Doing chores around the house; PF7 = Having aches or pains; PF8 = Low energy levels). % Percentage of improvement comparing Post versus Pre. Wilcoxon ranks test:

* *p* < .05 indicates significant improvement compared to the pre-intervention assessment (*p* = .012, *z* = − 2.527)

**Table 4:** PedsQL pre-post: Psychosocial Health Summary

Patient ID	SF			EF			SC		
	Pre	Post	%	Pre	Post	%	Pre	Post	%
Pt 1	75	85	13.33	60	95	58.33	70	70	0.00
Pt 2	55	80	45.45	55	75	36.36	40	70	75.00
Pt 3	40	65	62.50	45	55	22.22	50	70	40.00
Pt 4	75	90	20.00	85	75	− 11.76	65	75	15.38
Pt 5	45	60	33.33	80	85	6.25	65	70	7.69
Pt 6	35	40	14.29	30	75	150.00	10	45	350.00
Pt 7	85	100	17.65	100	100	0.00	90	95	5.56
Pt 8	85	90	5.88	70	100	42.86	55	80	45.45
All	60.60	76.30*	25.91	65.60	82.50*	25.76	55.60	71.90*	29.32

Psychosocial Health Summary: *SF* Social functioning scale score (items: SF1 = Getting on with others; SF2 = Children not wanting to be friends; SF3 = Teased; SF4 = Not able to do the same things; SF5 = Keeping up); EF = Emotional Functioning Scale Score (items: EF1 = Afraid/scared; EF2 = Sad; EF3 = Angry; EF4 = Sleeping; EF5 = Worrying); School Functioning Scale Score (items: ScF1 = Paying attention; ScF2 = Forgetting; ScF3 = Keeping up; ScF4 = Missing school—unwell; ScF5 = Missing school—appointments). % Percentage of improvement comparing Post versus Pre. Wilcoxon ranks test:

* *p* < .05 indicates significant improvement compared to the pre-intervention assessment (SF: *p* = .035, *z* = − 2.11; EF: *p* = .011, *z* = − 2.54; SC: *p* = .018, *z* = − 2.37)

**Table 5:** Descriptive results interview quantitative questions to the patients

IVR experience questions	*N*	*M*	ST	Range	Minimum	Maximum
How easy was it to set up the game?	7	9.29	0.95	2	8	10
How difficult was it to use the game?	6	3.17	1.60	4	0	4
How painful was it to use the game?	6	1.33	1.75	4	0	4
How much did you enjoy the game?	7	9.00	1.29	3	7	10

*M* Mean, *ST* Standard deviation. Answer range from 0 (nothing) to 10 (much). Patient 5 could not participate in the interview

**Table 6:** Data extracts: General impressions

Participant ID	Page	Line number	Quotation
Pr 1	3	1122-125	I think it is brilliant and hope that it is gonna help a lot of other children in physio
Pr 8	30	1221	I think it could be a really good step forward
OT	3	83–85	All the participants have said that they have enjoyed it and that they liked the game and that they find it beneficial
		97–99	I think I really good result from the people that have taken part and really good feedback, so I think it would be really useful to use in other areas
		102–103	I think a lot of the patients have said that if it comes back, they had love to try it again

*Pr* Parent, *OT* Occupational therapist

**Table 7:** Data extracts: System Playability: Learning and Mastery

Participant ID	Page	Line number	Quotation
Pt 2			We had a bit of trouble finding the game at first, but once we found it, it was pretty easy
Pt 4 (14y)	15	603–605	I am kind of confused and that as well because you need to learn how to start it up on that, but then after they [OT] do that, it is like I find it easier to use and set it up
	16	638	I had no problems playing that at home
Pt 5 (15y)	20	796	It was pretty spot on. I knew exactly what to do
		805–806	[first-time use] It was a bit weird, but it was nothing you cannot get used to
		818, 819	There were a couple of times where I forgot what to click, but that was it
Pr 1	1	32–37	The only one thing that she (patient) was unsure about was that she needed to progress the levels in order to go onto the climbing ones. She did not realise that she had to do that. She thought she had a different option to go on different games, but it was all linked together
Pr 3	10	429	I think like most things, it was easier once we got home and we had a chance to get used to it
Pr 7	26	1055–1057	(future deployment) put out different levels with the different ages, because I think with VR games and computer games, there is quite a big difference from, say, a seven-year-old to a 16-year-old

*Pt* Patient, *Pr* Parent

**Table 8:** Data Extracts: Comparison of IVR with CR: Natural movements

Participant ID	Page	Line number	Quotation
Pt 1	2	84	It was really easy to move my arm, and I did not realise
Pt 3	11	479, 481–482	You did not feel like you were doing physio. It just felt like you were playing a game
Pt 4	15	613–614	It felt good because you are like in your own […] makes you like happy and concentrating on the game
Pr 1	3	85–88	The range of movements seemed to be a lot more fluent. It was just like, it was a natural reaction to just do that, and the movement was just better than it would be if you had not had it [VR headset] on
		101–103	I think is a much more fun and encouraging way to do that for children without them subconsciously thinking about what they are doing
		114–117	[reproduction of movement with the VR] It just felt as though it was like I say a natural reaction for her [the patient] to do it. And did not look as though it was hard work for her to hold. And she was not complaining of any pain or uncomfortableness during. So to me, it was really beneficial for her
Pr 2	7	313–318	[Patient name] is quite healed now. So when we went through physio, she struggled doing the movements because it hurt so much. But if we had the game, I think she had just done it without realising part of the time
	9	380–384	When she was doing her physio, she was reluctant to do it because she knew It was going to hurt, and she knew what was coming. So, the game, it just does not make them think about it. They just do it. Because he is not thinking she needs to be doing the physio, like normal he is doing, yeah
	8	338–339	Her measurement moving back, so that was a really positive come out of it
Pr 3	11	487–494	She has been doing physio in the past, and it is been, like, do this exercise so many times a day for several minutes. And it is a chore. And you know, she will do this and then lose interest, forget that day or could not or whatever. The fact it was a game and she found it engaging and enjoyed it meant she looked forward to doing it every day. And I think that because it was more entertaining, she was doing the exercises without realising she was doing it
Pr 5	21	872–873	When I was watching him play the game, I saw the ease of movement really good for his rehabilitation on his arm
Pr 8	28	1214–1218	I feel like she was getting more out of it, she was using her arm more because subconsciously does not think about it, do you when you are doing something like that. And I think that it is definitely made her realise what she can do with it [frame] on, and it is made her movements a lot better

*Pt* Patient, *Pr* Parent

**Table 9:** Data extracts: Comparison of IVR with CR: Motivation

Participant ID	Page	Line number	Quotation
Pt 8	28	1144	It [IVR] was more fun than I wanted to do it all
Pr 1	4	180–182	When they[child] go to physio, they do not always see how much they are progressing. They see it as a long process where with that it is more of a fun way and it is a bit of a distraction of the overall goal really
Pr 3	13	575–578	She[child] was so engaged with it and so, sort of, looking forward to doing it every day. She did not need a prompting…it was not, oh come on now, you need to do your exercises. She made time every day herself to do it because she actually ends up doing it
Pr 4	19	765–768	I wll say the first operation, he is not enjoying the physio. He sometimes even mention to me he did not want it doing. But right now, when he is using the headset, when he is back to school, every day he is taking. “Come mum, I am ready for the video”
Pr 5	22	906–907	He was doing more. At one stage, he actually came to me and said “I will go on that VR now, mum” without me saying “Are you going on the VR?”
		923–924	I think that it makes it fun, and it makes them want to do it […] it does not come across as chore
Pr 7	26	1048–1050	They[child] are engaged in the game as opposed to doing the exercises so they are quite boring for children
Pr 8	30	1212–1214	I think at the start it took a lot of responsibility off me because I were not having to remind her or support her as much, you know, doing physio
OT	2	56 – 62	I think that like we’ve had some patients that are not very compliant with therapy or do not engage or do not complete the exercises that we gave them, which I understand because it is an extra thing to add onto their day, and that their movement had improved when they had come back because it was something they are engaging in and wanted to do every day. So, yeah, I think that it definitely had a good impact on people’s motivation to do the therapy, a lot more fun
		78–80	If it the patient that is not engaging well, it could take it home for a week or two and that improves that movement at home doing something that they want to do

*Pt* Patient, *Pr* Parent, *OT* Occupational therapist

## Data Availability

The approvals from Sheffield Hallam University and the National Health Services restrict us from sharing any raw data.
